# Map of physical interactions between extracellular domains of Arabidopsis leucine-rich repeat receptor kinases

**DOI:** 10.1038/sdata.2019.25

**Published:** 2019-02-26

**Authors:** G. Adam Mott, Elwira Smakowska-Luzan, Asher Pasha, Katarzyna Parys, Timothy C. Howton, Jana Neuhold, Anita Lehner, Karin Grünwald, Peggy Stolt-Bergner, Nicholas J. Provart, M. Shahid Mukhtar, Darrell Desveaux, David S. Guttman, Youssef Belkhadir

**Affiliations:** 1 Department of Biology, University of Toronto – Scarborough, 1265 Military Trail, Toronto, Ontario, Canada; 2 Department of Cell & Systems Biology, University of Toronto, 25 Willcocks St., Toronto, Ontario, Canada; 3 Gregor Mendel Institute (GMI), Austrian Academy of Sciences, Vienna Biocenter (VBC), Dr Bohr Gasse 3, Vienna, 1030, Austria; 4 Department of Biology, University of Alabama at Birmingham, Birmingham, Alabama, USA; 5 Protein Technologies Facility, Vienna Biocenter Core Facilities (VBCF), Vienna, Austria; 6 Centre for the Analysis of Genome Evolution & Function, 25 Willcocks St., University of Toronto, Toronto, Ontario, Canada

**Keywords:** Plant signalling, Plant stress responses, Plant molecular biology

## Abstract

Plants use surface receptors to perceive information about many aspects of their local environment. These receptors physically interact to form both steady state and signalling competent complexes. The signalling events downstream of receptor activation impact both plant developmental and immune responses. Here, we present a comprehensive study of the physical interactions between the extracellular domains of leucine-rich repeat receptor kinases (LRR-RKs) in Arabidopsis. Using a sensitized assay, we tested reciprocal interactions among 200 of the 225 Arabidopsis LRR-RKs for a total search space of 40,000 interactions. Applying a stringent statistical cut-off and requiring that interactions performed well in both bait-prey and prey-bait orientations resulted in a high-confidence set of 567 bidirectional interactions. Additionally, we identified a total of 2,586 unidirectional interactions, which passed our stringent statistical cut-off in only one orientation. These datasets will guide further investigation into the regulatory roles of LRR-RKs in plant developmental and immune signalling decisions.

## Background & Summary

Due to their sessile nature, plants must be able to accurately sense ever-changing signals from their local growth environment. The detection of environmental signals at the cell surface is mediated partly through the molecular activities of an expanded protein family of leucine-rich repeat receptor kinases (LRR-RKs), with 225 members in *Arabidopsis thaliana* (hereafter Arabidopsis)^1,2^. This family of receptors controls key developmental and immune responses^3,4^. In spite of great efforts, the vast majority of these proteins still have no defined biological functions, and therefore little progress has been made in understanding how the signals downstream of these receptors are integrated to guide decisions that regulate both plant development and pathogen defense processes^5^.

The best-studied examples of LRR-RK activation initially involve a ligand binding to the extracellular domain (ECD) of a cognate receptor. This induces the physical interaction between the ECDs of the receptor LRR-RK and a co-receptor LRR-RK to form a signalling competent complex^4^. The identification of shared components between immune and developmental signalling complexes provides at least one possible mechanism for the observed cross-talk between these pathways^6^. Systematic information about how members of the LRR-RK family physically interact to affect signalling has not been previously available, as these proteins are biochemically poorly tractable. To systematically identify the physical interactions between the ECDs of LRR-RKs in the model plant Arabidopsis, we undertook a large-scale screening effort. The interactions between LRR-RKs are known to be transient and of low affinity, especially in the absence of an activating ligand, and we therefore implemented the sensitized extracellular interaction assay (ECIA) method to test for interactions^7^. The method is based on the avidity-based extracellular interaction screen (AVEXIS) technique, which has been optimized to observe weak interactions. In AVEXIS the prey protein construct includes a pentamerization domain to increase assay sensitivity^8^. In a recent publication, we cloned and expressed the ECDs of 200 of the 225 Arabidopsis LRR-RKs in both bait and prey constructs to conduct an all-by-all protein interaction screen, and thus assayed the total possible LRR-RK interaction space to a 79% completeness^9^.

Here, we present the data from that study in its most expanded form, including the data used for the published analyses, an additional analysis that identifies a set of interactions that are found in only one orientation, and the raw data needed for the implementation of other normalization and hit-calling protocols. These data provide unique opportunities to formulate experimentally testable hypotheses aimed at understanding further how physical interactions in LRR-RK complexes control plant developmental and immune responses. We chose an extremely stringent cut-off to build a high-confidence interaction network including the most reliable bidirectional interactions. Next, we used these data to: i- assign biological function to previously uncharacterized receptors, and ii- demonstrate that the interconnectivity of physical interactions between LRR-RKs is a requisite to appropriately transduce a complex range of environmental signals to the plant^9^. However, the use of such stringent statistical cut-offs to produce the bidirectional dataset has likely resulted in the omission of biologically relevant data. For instance, interactions occurring only in one of the bait-prey or prey-bait orientations (unidirectional) have the potential to yield further biological insights.

## Methods

The methods described here are expanded from those found in our related work on this topic^9^.

### Expression of the extracellular domains of LRR-RKs.

The ECDs of LRR-RKs present several challenges for effective expression, which has led to a dearth of studies involving the use of recombinant proteins on a large scale. To express these domains, we first identified the location of signal peptides and transmembrane domains to determine the boundaries of the extracellular domains. The signal peptide was identified using SignalP4.0^10^, and the transmembrane domain predicted using Phobius^11^, TMHMM^12^, and other prediction programs for secondary structure prediction such as InterPro^13^. We further improved ECD boundary prediction by visual inspection of primary amino acid sequences to identify the location of the N- and C-terminal cysteine-capping consensus motifs (CXXXXC and variations thereof). The LRR domains form a hydrophobic core and these motifs are thought to cap this region and produce disulphide bonds to maintain proper tertiary structure. We have found that removal of these cysteine caps results in reduced expression and solubility *in vitro*. Once the proper sequence was determined, we designed primers that added the additional sequences required for RecA-mediated Sequence and Ligation Independent Cloning (Supplementary Table 1). Amplification was done using Phusion Flash Mastermix (Thermo Scientific) according to the manufacturer’s instructions for 2-step Polymerase Chain Reaction. 176 ECDs were cloned from the plasmid templates available from the Arabidopsis Biological Resource Center^14^, while the remaining 24 were cloned from Arabidopsis seedlings and mature leaves using RT–PCR. The amplified sequences were inserted into the pECIA-2 (for expression as bait) and the pECIA-14 (for expression as a prey) vectors for expression in *Drosophila melanogaster* Schneider 2 (S2) cells (vectors were a gift from C. K. Garcia)^7^. These expression vectors are modified versions of pMT/BiP/V5 (Invitrogen, V4130-20), which are driven by a copper-inducible *Drosophila* metallothionein promoter and contain the *Drosophila* BiP protein signal sequence. Sequences were cloned between the existing BiP signal sequence and the C-terminal epitope tags specific to each vector, and the presence of the correct ECD insert was confirmed with Sanger sequencing prior to expression.

### LRR-RK extracellular domain expression

All proteins were expressed using *Drosophila* S2 cells cultured at 27 °C in ESF 921 Insect Cell Culture Medium, Protein Free (Expression Systems). S2 cells were transiently transfected with expression vectors using Effectene (Qiagen) per manufacturer’s instructions followed by incubation at 21 °C. Twenty-four hours later, protein expression was induced with 1 mM CuSO_4_ and the supernatant was collected after three days of induction. After harvest the media containing the expressed ECDs was supplemented with protease inhibitor cocktail (Sigma) and 0.02% NaN_3_ and then stored at 4 °C until use. Protein expression was assessed by western blotting using anti-V5 antibodies (Invitrogen) for bait proteins or by alkaline phosphatase activity quantification for prey proteins.

### Primary reciprocal interaction screen

Pairwise reciprocal interaction assays were performed largely as previously described for the extracellular interactome assay with the modifications noted below^7^. The media containing the recombinant ECDs was diluted four-fold with PBS buffer containing 1 mM CaCl_2_, 1 mM MgCl_2_ (equilibration buffer), and 0.1% bovine serum albumin (BSA; Sigma). First, assay plates were prepared by adsorbing 100 μl of media containing bait proteins fused with an Fc domain, directly to 96-well protein-A-coated plates (Thermo Fisher Scientific) by overnight incubation at 4 °C. The coated plates were then washed with a PBS solution containing 0.1% Tween-20 before use to remove any loose protein that could interfere with subsequent protein interaction. The washed plates were then blocked with 100 μl of equilibration buffer containing 1% BSA for 3 h at 4 °C and then washed once more. 100 μl of the diluted media containing the prey proteins fused to alkaline phosphatase was then added to the wells and incubated for 2 h at 4 °C and then washed away prior to adding the alkaline phosphatase (AP) substrate (KPL). Protein interaction between bait and prey was quantified by measuring the absorbance at 650 nm using a Synergy H4 Multi-Mode plate reader (BioTek) after 2 h of incubation at room temperature. In addition to quantitative data, an image was captured of each 96-well plate for visual inspection. Visual inspection ensured that the included positive (containing the known interaction pair BAK1-BIR4^15,16^) and negative (prey only) control wells performed as expected, allowing the associated plate to be included in downstream analysis. The complete set of raw absorbance values was combined into a binary dataset using an in-house designed script (Platero v0.1.4), and then subjected to post-experimental statistical analysis to remove both false positive and false negative interactions.

### Data analysis

The complete set of raw absorbance values (Data Citation 1) for each protein pair in both directions was compiled into a data matrix containing 200 columns and 200 rows. To accurately compare absorbance values, we first needed to eliminate any bias in the data arising from the differential background binding capacities of the individual bait and prey protein preparations. This may arise due to variation in protein expression, protein stability, quality of Protein A coated plates, or intrinsic ‘stickiness’ of certain proteins. We implemented a two-way median polish approach to remove any effects that arose as a result of specific protein preparations^17,18^. In the data matrix, each row represented an individual bait preparation, and each column represented an individual prey protein preparation. The 2-way median polish has the effect of removing first any assay wide effect (the background level of absorbance in the assay and any effect of the 96-well plates used), followed by removing any row or column specific effects (e.g. the intrinsic level of absorbance associated with a given protein preparation). The result is a data matrix containing only the residual values, which in effect are the amount of the observed absorbance that can be attributed to the physical interaction between the bait and prey having considered the overall level of absorbance associated with those proteins screen-wide. Thus, those protein pairs that physically interact can be viewed as outliers in the dataset, regardless of whether the individual proteins involved show an overall high or low level of background absorbance in the screen.

The calculated residuals were then used to identify true interactions, which appear as outliers in the data distribution. We hypothesized that the data should contain mainly protein pairs that show no physical interaction, with a small subset of pairs that show high levels of interaction. This would result in a unimodal distribution centred on 0 with a tail to the right containing the interactions. Therefore, any statistical methods we use must be robust to outliers. We chose to use the median and median absolute deviation (MAD), rather than mean and standard deviation, to score the screen for this reason. The MAD is the median of the absolute deviation of each data point from the overall data median. After calculating the MAD value for the data, we can then relate each individual residual measurement in terms of the number of MAD units that it is away from the median value.

We chose an arbitrary, yet widely used, score cut-off of 2.5 to identify high confidence interactions, which results in a set of 2586 protein pairs that interact in one orientation (6.5% of the 40,000 interactions tested, Data Citation 2). In addition, we determined that 567 interactions were found in both the bait-prey and the prey-bait orientations (2.8% of the 20,100 bidirectional interactions tested including self-interactions, which are effectively tested in both directions in a single well, Data Citation 3). To isolate these bidirectional interactions, we chose to use the geometric mean of the scores of the interaction as measured in the bait–prey and prey–bait orientations to avoid any undue influence by extreme values. Any value for which the geometric mean product was greater than 2.5 was considered significant for the highest-quality, bidirectional dataset (Data Citation 3). These two datasets are publicly available online at the Bio-Analytic Resource (BAR) under accession (MI 2189, Smakowska-Luzan *et al.*^9^, doi:10.1038/nature25184): http://bar.utoronto.ca/interactions.

### Confirmation test of the predicted interactions

To test the accuracy of our predicted physical interactions, we selected all 567 bidirectional interactions and a random subset of the non-interacting pairs for independent retest. For each of the interactions retested, the ECDs were newly re-expressed and retested in both the bait–prey and the prey-bait orientations. To ensure there was no non-specific binding to the plates, we included three prey-only negative control wells for each interaction tested. To ensure that the assay functioned as expected each plate also contained a positive control well containing the known interacting proteins BAK1 and BIR4.

To validate the predicted interactions identified in the primary screen, the data from the primary and retest screens first had to be made directly comparable. As the primary screen scoring methods rely on a large and unbiased population containing mostly non-interacting protein pairs, we were unable to use the same procedure for the retest data, which is small, biased in its composition, and contains a majority of predicted positive interactions. Instead, we implemented a multi-stage hit calling method to ensure reliable data confirmation. To ensure that the retest and primary screen data could be directly compared, the absorbance values from the two datasets for each interaction were paired and the data subjected to an interquartile range (IQR) normalization step. This results in scaled data that can be directly compared. For both datasets the geometric mean absorbance for each bidirectional interaction was calculated using the bait-prey and prey-bait values. To identify an appropriate cut-off for identification of positive physical interactions, the threshold geometric mean value for inclusion in the bidirectional dataset in the primary screen was calculated and found to be 0.090989. Therefore, any interaction in the retest screen with a geometric mean value > 0.090989 was considered positive, while all others were considered negative.

### Code availability

The custom PLATERO script used for concatenating the interaction absorbance values is available from https://github.com/AdamMott/platero-code.

## Data Records

The raw interaction absorbance data from the CSI has been deposited to FigShare (Data Citation 1), and has previously been available in Supplementary Table 11 of Smakowska-Luzan *et al.*^9^. The column headings are as follows:

### Bait_ECD

This is the unique locus identifier for the Arabidopsis gene from which the nucleic acid sequence corresponding to the ECD was extracted and cloned into the bait expression plasmid.

### Prey_ECD

This is the unique locus identifier for the Arabidopsis gene from which the nucleic acid sequence corresponding to the ECD was extracted and cloned into the prey expression plasmid.

### Abs_650_nm

The raw absorbance value measured in the primary screen for the bait-prey pair listed.

The protein interactions from this publication have been submitted to the IMEx (http://www.imexconsortium.org) consortium through IntAct^19^ and assigned the identifier IM-26261 (Data Citation 2), while those described in Smakowska-Luzan *et al.*^9^ can be found in IM-26260 (Data Citation 3).

## Technical Validation

The technical validation of the interaction data is presented in detail in Extended Data Figures 1–3 of Smakowska-Luzan *et al.*^9^. In brief, the successful expression of each construct was first verified by western blot performed either on the S2 cell media or after purification on protein-A coated 96-well plates. Then, we used the known interacting proteins FLS2 and BAK1 to optimize assay conditions. We ensured that the protein concentrations contained in the media were sufficient to measure interaction in a ligand-independent manner due to the sensitized nature of the assay. At sub-optimal protein conditions, we demonstrate that the interaction can be made ligand-dependent, demonstrating that the expressed proteins functioned as expected. We further optimized the temperature of protein production and the pH of the assay to maximize the sensitivity of the interaction assay.

After completing the primary screen, we next tested the reliability and reproducibility of the identified interactions. To address reproducibility, we compared the values calculated from the primary and retest screens to each other and to the pooled negative control measurements from both screens, which are composed of prey only wells (n = 618). As expected, the non-interacting protein pair data distributions from both the primary and retest screens were statistically indistinguishable from the pooled negative controls, while the bidirectional interaction sets were statistically significantly different from all negative sets. We also directly compared the values from the primary screen and retest, which showed a Spearman correlation coefficient of 0.769, further demonstrating the reproducibility and reliability of the interactions measured in the screen.

## Usage Notes

There are numerous potential uses for the presented datasets. First, direct physical interaction with a receptor of known biological function is a valuable predictor of function for uncharacterized receptors. Thus, the data can be used as a powerful hypothesis generation tool for subsequent experiments to identify novel actors in well-studied biological processes.

Second, the dataset as a whole can be used to construct a network analysis of the interactions, as we demonstrated in our recent publication^9^. Our network analysis is only one such representation, and similar networks could be built using different input data arising from the unidirectional interaction set, or from novel analyses of the raw data. A novel analysis of the network structure in fact has recently been used to derive powerful insights into how pathogens target the network to subvert plant immunity^20^. Such network structures are excellent tools for further inspection of other publicly available datasets, such as mapping gene expression data onto the network to identify co-expressed or co-regulated sub-networks.

## Additional information

**How to cite this article**: Mott, G. A. *et al*. Map of physical interactions between extracellular domains of Arabidopsis leucine-rich repeat receptor kinases. *Sci. Data*. 6:190025 https://doi.org/10.1038/sdata.2019.25 (2019).

**Publisher’s note**: Springer Nature remains neutral with regard to jurisdictional claims in published maps and institutional affiliations.

## Supplementary Material



Supplementary Table 1
